# Supplementary material for: Effect of the COVID-19 lockdown on the HIV care continuum in Southwestern Uganda: A time series analysis
Source: PLoS One. 2023 Aug 10;18(8):e0289000. doi: 10.1371/journal.pone.0289000 (PMC10414556; doi:10.1371/journal.pone.0289000)
Supplement: S1 Appendix — (DOCX) [file pone.0289000.s001.docx]

**Supplementary Appendix 1.** Alternative model specification using autoregressive integrated moving average (ARIMA) model.

Sensitivity analyses were conducted to test whether alternative model specifications would have substantially changed our effect estimates. An assumption of standard regression models, including Poisson regression, is that observations are independent.^1^ This assumption is more likely violated in time series data, given the potential of autocorrelated outcomes. Thus, analyses were re-run by fitting dynamic harmonic regression models with ARIMA errors, as previously described.^2,3^

In the figures on the next page, we present the observed trend (in black) and expected trends using Poisson or fractional probit regression with calendar fixed effects (in red; primary model) and dynamic harmonic regression models with ARIMA errors (in blue). Overall, expected trends were similar between the two models, except for two outcomes; ARIMA models suggest COVID-19 was associated with a greater reduction in ART initiations and more missed visits in Mbarara Referral Hospital compared to our current approach. However, the ARIMA model predicted ART initiations would increase post-COVID-19 in Masaka Referral Hospital, despite observing a long-term decreasing trend during the pre-COVID period. Thus, we believe our primary model estimates of the ‘expected trend’ seem more plausible than predictions from the ARIMA model. Similarly, the ARIMA model predicted the proportion of missed visits would have reduced or remained stable during the post-COVID-19 period, despite observing a modest increasing trend during the pre-COVID-era. This finding could be plausible as we see that the proportion of missed visits remained stable during the 2018-2020 period (pre-COVID). Thus, these estimates should be interpreted with caution.

**References**

1. Bernal JL, Cummins S, Gasparrini A. Interrupted time series regression for the evaluation of public health interventions: a tutorial. *Int J Epidemiol* 2017; **46**(1): 348-55.

2. Hyndman RJ, Athanasopoulos G. Forecasting: principles and practice: OTexts; 2018.

3. Chen Y-H, Glymour MM, Catalano R, et al. Excess mortality in California during the coronavirus disease 2019 pandemic, March to August 2020. *JAMA Intern Med* 2021; **181**(5): 705-7.
