# Supplementary material for: Effect of the COVID-19 lockdown on the HIV care continuum in Southwestern Uganda: A time series analysis
Source: PLoS One. 2023 Aug 10;18(8):e0289000. doi: 10.1371/journal.pone.0289000 (PMC10414556; doi:10.1371/journal.pone.0289000)
Supplement: Attachment — Submitted filename: Response to Reviewers 15Mar2023.docx [file pone.0289000.s003.docx]

**15^th^ March 2023**

**Title: Effect of the COVID-19 lockdown on the HIV care continuum in Southwestern Uganda: An interrupted time series analysis**

**Manuscript Number: PONE-D-22-34356**

To PLoS ONE,

Please find below responses to the reviewer’s comments detailed below.

Reviewer #1: Overall, this is a clearly-written and straightforward manuscript that presents relevant data on the impact of the COVID-19 pandemic lockdowns on engagement in HIV care. The findings are timely and relevant both to the literature examining the impact of lockdowns as well as how to best deliver care in the early differentiated service delivery. I have several specific critiques and a few minor points:

1. The authors’ choice to examine two separate lockdowns (April-May 2020 and June-August 2021), in addition to the “overall” period of April 2020 - September 2021, is confusing. The scientific rationale for examining the “overall” period in addition to the actual lockdowns is unclear. Additionally, it is not obvious from reading the manuscript whether they actually conducted three separate interrupted time series analyses (comparing baseline to each of these periods), or somehow incorporated all of the time periods into a single analysis.

RESPONSE: Thank you for the comment, first we evaluated the effect of lockdown on HIV outcomes by comparing the pre-lockdown to the overall lockdown period. However, more strict lockdown policies were implemented in response to surges in COVID-19 cases and deaths. We had hypothesized that effects would change as the restrictions were gradually relaxed so we wanted to assess those trends.

2. The segmented regression model correctly includes coefficients for linear time and time since the start of the first lockdown, but I believe that it should also include a non-linear coefficient indicating whether the period is prior to or during lockdown (See Jo Y, et al JIAS 2021, or Ross J, et al JIAS 2019).

RESPONSE: We thank the reviewer for this comment and agree that inclusion of this coefficient is necessary for many interrupted time series analyses where the intervention had a sudden and substantial impact on the outcome (described as a level change in the Ross et al, JIAS 2019 manuscript). However, we did not observe this in our study and thus, decided to exclude this coefficient. Despite not including this coefficient, we believe that our impact estimates of the lockdown would have remained the same, as our estimates of the expected trends were derived from models that used pre-lockdown outcomes only and impact estimates were calculated based on the difference between expected and observed trends.

3. What was the rationale for stratifying the analysis by the site (Mbarara and Masaka)? Table 1 suggests that the populations were quite similar, and presumably they experienced lockdowns at the same time. So why not combine the data?

RESPONSE: Thank you for the comment. The two sites are supported different implementing partners so we anticipated that the adaptations might be different. Additionally, the viral suppression rates differed by study site, so we decided to present the data separately.

4. In the Discussion, the authors do a nice job describing steps that the Ugandan HIV program took to counter potential effects of the lockdown on missed visits (e.g. Community Distribution Points, multi-month dispensing), which may have contributed to relatively few missed visits. However, I wonder if the authors also looked at the metric of number of *scheduled* visits in addition to missed visits. It would be useful to know whether fewer visits occurred overall during the lockdowns - if this was the case, but clinical outcomes were not impacted, this would help make a case for lower-barrier DSD models.

RESPONSE: Thank you for raising this concern. The missed visits were computed basing on the appointments (scheduled visits). During the lockdown, scheduling of visits for PWH was not interrupted. Patients who could not make it to the clinic for the visit were advised to seek care from nearby health facilities. However, data for these patients were still captured in the database. We include this in the discussion section page, line, as a possible explanation as to why there was no significant effect of the lockdown on missed visits (page 15, line 257)

5. Similarly, it is promising to see high levels of viral suppression, but it is possible that a substantially fewer number of people had a viral load *measured* during the lockdown periods. It would be helpful for the authors to present data on the number of viral loads in addition to the proportion suppressed.

RESPONSE: Thank you for raising this concern. We have noted this limitation and added it to the discussion section as follows “Third, we examined the effect of the lockdown on viral suppression but did not assess the effect on viral load coverage” (page 18, lines 282-284)

Minor comments:
6. Intro, paragraph 2, line 73: Can the authors briefly note the findings of the cited studies (references 7 and 11)?

RESPONSE: Thank you, we have included the findings from the two studies as follows “In Uganda, studies conducted to evaluate the effect of the lockdown on HIV care showed an increase in missed visits by 15-50% and no effect on viral suppression” (page 5, lines 74-75)

7. The authors should not refer to patients as “participants” in the manuscript, as they utilized routine clinical data with a waiver of informed consent.

RESPONSE: Thank you for the comment. We appreciate your feedback. We have revised the word “participants” to Persons living with HIV (PWH) in all areas of the manuscript

8. In the Methods the authors should describe/define all variables that were extracted from the EMR (e.g. sex, age, marital status) in addition to outcomes.

RESPONSE: Thank you for the comment. We now include all the variables that we extracted from the EMR in the methods section (page 6, lines 109-110).

9. In Table 1, was the mean number of ART clinic visits extracted over the entirety of the study, or some sort of baseline measurement? If this was over the entire study period, as noted above, would be helpful to see the mean number pre-lockdown and then during the lockdown. Similarly, was duration on ART a baseline value?

**RESPONSE**: Thank you for this comment. We considered the mean number of ART clinic visits and mean ART duration for the entire study period. We considered a cohort of participants who initiated into care from March 2017 to September 2021. We have revised Table.1 to indicate the pre- and post-lockdown values as well and included a footnote for duration of ART describing that it was for the entire study period.

10. In Table 2, it would be helpful to have a footnote for “Expected” indicating that this value was derived from the linear regression model

**RESPONSE:** Thank you for your helpful suggestion, we have agreed to the suggestion and added the footnote “*Expected” on Table 2, page 10, line 185

11. The authors state that all data is available but this does not seem to be the case.

**RESPONSE**: Data is available on request by the reviewer. Due to the participant privacy policy and use of patient data , we are able to provide access link of the data to the reviewer and PLOS ONE team when requested as opposed to making it available to the repository

12. Reviewer #2: Thank you for the opportunity to review this well written manuscript. The study design and analysis approach is appropriate. The ethical considerations were made. The authors have endeavoured to provide contextual information on how the lockdowns were implemented, the study setting and the type of HIV program innovations which are all critical in understanding the findings of this study. The study strengths and limitations are described.

13. The only question that remains is how other facilities, which are not part of the IeDEA collaboration, would perform with regard to implementing innovations for retention for people living with HIV/AIDS.

**RESPONSE**: Thank you for highlighting this limitation which we have now clarified under the discussion section to include similar adaptations described in none IeDEA low level facilities page 17/line 287-288
