# Supplementary material for: Effect of the COVID-19 lockdown on the HIV care continuum in Southwestern Uganda: A time series analysis
Source: PLoS One. 2023 Aug 10;18(8):e0289000. doi: 10.1371/journal.pone.0289000 (PMC10414556; doi:10.1371/journal.pone.0289000)
Supplement: Attachment — Submitted filename: Response to Reviewers.docx [file pone.0289000.s004.docx]

**Title: Effect of the COVID-19 lockdown on the HIV care continuum in Southwestern Uganda: An interrupted time series analysis**

**Manuscript Number: PONE-D-22-34356**

Reviewer #1: The authors were responsive to a number of critiques in their revision and the manuscript is improved. However, I do not feel that they adequately responded to several major critiques:

1. The authors have clarified that they examined three different policy periods (overall lockdown, first intense lockdown, second intense lockdown) because of the intensity of the restrictions. However, it remains unclear how this was incorporated into the analysis, as the segmented regression model only includes a term for post overall lockdown period, and no term for the more intense/shorter lockdowns.

**RESPONSE:** We agree with the reviewer and have added level change and slope change terms to our ITS models to allow for a more accurate examination of the different policy periods. The Methods section has been edited to reflect the new model specification (in Page 8, lines 133-144 and provided below) and revisions have been made throughout the manuscript text, Tables, and Figures.

“The segmented regression model used to estimate pre- and post-trends was specified as follows: $Y_{t}=\beta_{0}+\beta_{1}T_{t}+ \beta_{2}X_{t}+ \beta_{3}A_{t}+\beta_{4}M_{t}+ \beta_{5}V_{t}+\beta_{6}C_{t}$, where for time points $t$, $Y$ represents the outcome; $T$ is a linear term indicating the number of months since the start of the study period (models the pre-lockdown trend); $X$ is a binary term indicating the period after the first lockdown (models level of change first lockdown); $A$ is a linear term indicating the number of months since the start of the first lockdown policy in April 2020 and values prior to April 2020 would be equal to zero (models the slope change of the first lockdown); $M$ is a binary term indicating the period after the second lockdown (models level-change of second lockdown); $V$ is a linear term indicating the number of months since the start of the second lockdown in June 2020 and values prior to June 2020 would be equal to zero (models the slope change of the second lockdown); and $C$ is an indicator variable for calendar month (e.g., January, February, March etc.) to account for seasonality.”

2. Another statistical concern - I am not sure that the justification for not including a level change coefficient in the segmented regression model is appropriate/sufficient. The manuscript would benefit from review by a statistician to ensure the analyses were performed correctly.

**RESPONSE:** Kindly see response to Reviewer #1, Comment #1.

3. The justification for analyzing Mbarara and Masaka separately is somewhat weak and is not described in the manuscript at all. At the very least, the authors could include this justification in the methods when describing how the analyses were performed.

**RESPONSE:** We thank the reviewer for this comment. We have provided the following justification in the Methods Section (Page 7, lines 122-126)

“Prior to conducting ITS analyses, we found substantial differences in pre-intervention outcomes (e.g., number of ART initiations, viral suppression rates) between study sites. Thus, ITS analyses were conducted separately for Mbarara and Masaka Referral Hospitals to explore whether effects of the lockdown varied between study sites.”
