# Supplementary material for: Effect of the COVID-19 lockdown on the HIV care continuum in Southwestern Uganda: A time series analysis
Source: PLoS One. 2023 Aug 10;18(8):e0289000. doi: 10.1371/journal.pone.0289000 (PMC10414556; doi:10.1371/journal.pone.0289000)
Supplement: Attachment — Submitted filename: Response to Editor Comments_YC.docx [file pone.0289000.s005.docx]

**Response to Editor’s Comments**

Please find below our response to the Editor’s comment. In our response, we have provided the **page and line numbers** where changes have been made to the manuscript.

**Comment:** We thank you for your second revision however, the response regarding the statistical soundness of the model has not been fully addressed. The authors indicate comparison by lockdown period however, the model does not appear to allow for such comparisons, per the previous reviewer comment. It might be reasonable to modify methods to indicate models across the lockdown periods however, further evidence that the proposed model allows for the comparisons across lockdown periods specified is needed, both here with the editors and to the readers in the manuscript. Many thanks for your time and we look forward to reviewing you next revision.

**Response:** We thank the editor and the reviewer for their comments regarding the proposed approach. To address these comments, we have revised our methods, updated our manuscript accordingly, and added sensitivity analyses using a different modelling approach. For these analyses, we sought out additional support from Dr. Yea-Hung Chen, who has expertise in epidemiology and biostatistics (particularly in methods used in this study) and has published several studies in high-impact journals utilizing time series analyses to determine the impact of COVID-19 on mortality^1-3^ and racial/ethnic disparities.^4^ Given Dr. Chen’s contributions to the analysis and overall writing of the manuscript, we kindly request he be added as a co-author to our study. In the following sections, we describe in detail our revised methods and the changes made to our manuscript.

In our revised manuscript, we have re-analyzed our data removing the use of segmented regression model. Rather, our study uses a different statistical approach to estimate the impact of the lockdown policies. Our assumption is that the underlying trend in the pre-intervention period would have continued had it not been ‘interrupted’ by the COVID-19 pandemic and its associated lockdown policies.^5-7^ The statistical model we used in our study utilized pre-intervention data only to establish the underlying pre-intervention trend and used this model to estimate the expected (counterfactual) trend during the post-intervention period. A graphical interpretation is provided below.

To establish the underlying trend, we modeled pre-intervention monthly data using Poisson regression, as done in previous studies.^8,9^ We included a continuous value indicating time in months and fixed terms indicating calendar months to account for seasonality. An alternative model specification was tested and included in the Supplementary Appendix. To estimate the impact of each post-lockdown policy period, observed outcomes during each post-lockdown month were compared to the expected (counterfactual) values predicted from the model and averaged across the post-lockdown period of interest (during the 1^st^ lockdown, during the 2^nd^ lockdown, and the overall 18-month period after the first lockdown). This described further in our revised Methods section (on **Pages 7-8; lines 121-142**).

The current analysis differs from our previous segmented regression approach in one major advantageous way. First, our current model only uses pre-intervention data to predict the expected ‘counterfactual’ trend during the post-lockdown period. Thus, the approach does not rely on modeling intervention-era time (or the policy context during that period). Thus, there is no need to incorporate any model covariates that indicate slope or intercept changes related to each lockdown policy (unlike with the segmented model approach). While this new approach does not evaluate these terms (i.e., slope or intercept changes), we argue that this is not strictly of interest. We are, rather, interested in the cumulative causal effect of the intervention.

While there is no single accepted statistical method for evaluating policy interventions,^8^ we recognize that the segmented regression approach is a most common and widely recognized approach.^5,10,11^ Thus, for clarity, we have **amended our manuscript throughout** to describe our study as a ‘time series’ analysis. Moreover, given the true counterfactual trend is unverifiable and estimated expected trends are dependent on the choice in statistical model, we added text in our Limitations section that estimates from our analyses should be interpreted with caution (**Pages 16-17; lines 275-282** and provided below):

“Third, impact estimates are highly dependent on the statistical model used to establish expected trend. We conducted sensitivity analyses using an ARIMA model which showed similar results for most outcomes, except for two outcomes; ARIMA models suggest COVID-19 was associated with a greater reduction in ART initiations in Masaka Referral Hospital and more missed visits in Mbarara Referral Hospital compared to our current approach. While we believe that our estimates of the expected trend are more plausible for reasons outlined in Supplementary Appendix 1, the estimates produced for these outcomes should be interpreted with caution (as the true counterfactual trend is unverifiable).”

**References**

1.	Chen Y-H, Matthay EC, Chen R, et al. Excess Mortality in California by Education During the COVID-19 Pandemic. *Am J Prev Med* 2022; **63**(5): 827-36.

2.	Riley AR, Chen Y-H, Matthay EC, et al. Excess mortality among Latino people in California during the COVID-19 pandemic. *SSM Popul Health* 2021; **15**: 100860.

3.	Chen Y-H, Glymour MM, Catalano R, et al. Excess mortality in California during the coronavirus disease 2019 pandemic, March to August 2020. *JAMA Intern Med* 2021; **181**(5): 705-7.

4.	Chen R, Aschmann HE, Chen Y-H, et al. Racial and ethnic disparities in estimated excess mortality from external causes in the US, March to December 2020. *JAMA Intern Med* 2022; **182**(7): 776-8.

5.	Bernal JL, Cummins S, Gasparrini A. Interrupted time series regression for the evaluation of public health interventions: a tutorial. *Int J Epidemiol* 2017; **46**(1): 348-55.

6.	Morgan SL, Winship C. Counterfactuals and causal inference: Cambridge University Press; 2015.

7.	Kontopantelis E, Doran T, Springate DA, Buchan I, Reeves D. Regression based quasi-experimental approach when randomisation is not an option: interrupted time series analysis. *BMJ* 2015; **350**.

8.	Harper S, Bruckner TA. Did the Great Recession increase suicides in the USA? Evidence from an interrupted time-series analysis. *Ann Epidemiol* 2017; **27**(7): 409-14. e6.

9.	Woolf SH, Chapman DA, Sabo RT, Weinberger DM, Hill L. Excess deaths from COVID-19 and other causes, March-April 2020. *JAMA* 2020; **324**(5): 510-3.

10.	Wagner AK, Soumerai SB, Zhang F, Ross‐Degnan D. Segmented regression analysis of interrupted time series studies in medication use research. *J Clin Pharm Ther* 2002; **27**(4): 299-309.

11.	Rossen LM, Branum AM, Ahmad FB, Sutton P, Anderson RN. Excess deaths associated with COVID-19, by age and race and ethnicity—United States, January 26–October 3, 2020. *MMWR* 2020; **69**(42): 1522.
